# Cognitive Performance in Euthymic Patients with Bipolar Disorder vs Healthy Controls: A Neuropsychological Investigation

**DOI:** 10.2174/1745017901713010071

**Published:** 2017-07-27

**Authors:** M. Carlotta Palazzo, Chiara Arici, Laura Cremaschi, Marta Cristoffanini, Cristina Dobrea, Bernardo Dell’Osso, A. Carlo Altamura

**Affiliations:** 1 Dipartimento di Fisiopatologia Medico-Chirurgica e dei Trapianti, Università degli Studi di Milano, Fondazione IRCCS Ca’ Granda, Ospedale Maggiore Policlinico, *via* Francesco Sforza 35, 20122 Milano, Italy; 2 Centro Sant’Ambrogio, Fondazione Sacro Cuore Fatebenefratelli, Provincia Lombardo Veneta, Cernusco sul Naviglio, Milano, Italy; 3 Dipartimento di Neuroscienze e Salute Mentale, Unità di Neuroradiologia. Fondazione IRCCS Ca’ Granda, Ospedale Maggiore Policlinico, *via* Francesco Sforza 35, 20122 Milano, Italy; 4 Department of Psychiatry and Behavioral Sciences, Bipolar Disorders Clinic, Stanford Medical School, Stanford University, CA, USA

**Keywords:** Bipolar Disorder (BD), Euthymia, Cognitive functioning, Bipolar subtypes

## Abstract

**Objectives:**

Cognitive impairment may affect patients with Bipolar Disorder (BD) beyond the acute episodes, qualifying as a potential endophenotype. However, which cognitive domains are specifically affected in euthymic patients with BD and the potential influence of confounding factors (*e.g.*, age and concomitant pharmacological treatment) are still a matter of debate. The present study was, therefore, conducted to assess cognitive performance across specific domains in euthymic bipolar patients, not older than 50 years (to avoid potential age-related bias) versus healthy controls (HCs).

**Methods:**

A cognitive task battery, including the Wisconsin Card Test, Span Attention Test, Tower of London, Trail Making Test, Verbal Fluency Test, Matrices Scores and N-Back, was administered to 62 subjects (30 bipolar patients and 32 matched HCs) and differences between the groups analyzed.

**Results:**

Bipolar patients performed significantly worse than HCs in the Span Forward task, in the expression of Verbal Fluency Test (Category) and in the N-Back task (all p<.05), with marginal differences between BD I and BD II patients.

**Conclusion:**

The present study pointed out significant differences in terms of cognitive performance between euthymic bipolar patients and HCs, supporting the notion that specific cognitive functions may remain impaired even after the resolution of the acute episodes in subjects suffering from BD. Future studies on larger samples are warranted to confirm the present results and further explore potential differences in cognitive impairment across specific bipolar subtypes.

## BACKGROUND

Bipolar Disorder (BD) is a highly disabling condition with a complex gene-environment etiology [1]. Among the core symptoms of BD, cognitive impairment seems to be a consistent feature during acute episodes [2, 3]. In particular, cognitive symptoms have been extensively investigated during depressive episodes, either in unipolar and bipolar patients [4]. For instance, attentional deficit, impaired short- and long-term memory, decision making and judgement have been reportedly observed in depressed patients and are among the criteria of Major Depressive Episode for DSM-5 [5] and ICD-10 [6]. Some studies point out that cognitive deficits represent an early feature of Major Depressive Disorder, with the involvement of executive functioning, attention, learning and memory, not only in the acute phase but throughout the entire course of the disease [7]. Data from the literature show that cognitive deficits can improve with pharmacological treatment [8, 9], but there is still uncertainty about which cognitive domains can be improved by treatment [10].

On the other hand, executive functions, memory, attention and psychomotor speed were found to be significantly altered in manic phases as well [11, 12]. In addition, cognitive impairment and general functioning were found to be deeply interconnected with a profound impact on clinical outcome [11, 12].

The hypothesis that bipolar patients – or at least a part of them - may present cognitive impairment beyond acute episodes has been supported by several reports and, more recently, meta-analytic studies [13-17]. In particular, the presence of cognitive impairment during euthymic periods was found to negatively influence patients’ functional outcome and global performance [18], like other well-established clinical features, such as the number of previous episodes [19] and the overall response to treatment [20].

Cognitive impairment, therefore, could be a specific trait of BD and some studies have associated altered brain activity, measured through imaging procedures, with cognitive deficits, suggesting that such domain may represent a heritable, susceptibility-related phenotype [21]. According to a recent meta-analysis, moreover, a different expression of cognitive dysfunction was reported in first-episode patients with BD [22] and in unaffected siblings of young bipolar patients, providing more direct support for its potential heritability [23]. Arts and colleagues [24], in a previous meta-analysis, indicated executive function and verbal memory as candidate bipolar endophenotypes, given the presence of important deficits in these domains in bipolar patients as well as the existence of small cognitive impairment in first-degree relatives. Follow-up studies on cognitive assessment of bipolar patients provide an additional confirmation in this respect. In fact, Arts and co-workers [25] evaluated the cognitive profile of a group of patients over a 2-year follow-up period and found that cognitive function varied significantly over time, with the exception of sustained attention that may represent an intermediary phenotype.

Even though cognitive functioning has been indicated as a promising endophenotype and a marker of global functioning/prognosis in psychiatric disorders, studies of cognitive performances in the field of BD showed mixed results. For instance, cognitive impairment was documented in specific areas, such as executive functions [26], memory [27, 28] and attention span/processing speed in patients with BD [29, 30]. However, it is unclear whether cognitive impairment is a general trait of BD (present, for instance, in BD I and BD II subjects), with investigation on potential differences between BD I vs II types substantially lacking [31, 32]. In addition, a large variety of cognitive tasks have been employed to assess cognitive impairment during euthymia, complicating the interpretation of results from different studies conducted with bipolar patients [33]. In fact, although cognitive impairment is recognized as an important clinical feature of BD, there is no standard cognitive battery that has been developed *ad hoc*. For instance, most neuropsychological batteries used to test patients with Schizophrenia were found to be as accurate for testing patients with BD as well [34].

Recently, it was shown that cognitive test measuring set shifting, inhibition/latency, verbal fluency and the ability of detecting differences and searching (*e.g.*, Trial Making Test, Stroop Color Word test) had the strongest ability to discriminate even subtle differences in the cognitive functioning of bipolar patients [33].

In light of the above discussion, the main aim of the present study was to explore cognitive performances in euthymic bipolar patients, using a specific cognitive battery, recognised in literature as reliable for assessing cognitive performance in psychiatric patients suffering from mood disorders [35]. In addition, the study was conducted on patients under the age of 50 years, in order to reduce potential age-related influence over cognitive functioning. Finally, we aimed to assess possible differences between patients suffering from BD type I vs type II, as this topic is less considered in literature.

## MATERIALS AND METHODS

### Sample

From an original sample of 65 bipolar patients, thirty patients with BD (15 patients with BD I and 15 with BD II) and 32 healthy controls (HCs) were recruited for the study. The main reasons for exclusion from the protocol were age > 50 years (90%) and mental retardation (10%). All participants were white Caucasian, who provided written informed consent, after receiving a complete description of the study, in accordance with the Declaration of Helsinki. The Structured Clinical Interviews for DSM-IV-TR [36-39] were used to confirm patients’ diagnosis and to exclude the presence of any Axis I and II disorder for healthy subjects. All patients and controls were recruited by psychiatrists working at the University Department of Mental Health at the Fondazione IRCCS Ca’ Granda, Ospedale Maggiore Policlinico of Milan, Italy. Additional exclusion criteria were: inability to give informed consent, mental retardation, a history of substance abuse within the past 6 months, the presence of any significant neurological and medical condition revealed by clinical and MRI evaluation. Lifetime psychiatric comorbidities were accepted, while cross-sectional ones were ruled out. When considering comorbidity, BD had to be the primary disorder, that is the one representing the main motivation to seek help and responsible for the most significant distress.

All patients were on stable pharmacological treatment with mood stabilizers (lithium, anticonvulsants and atypical antipsychotics) and, in some cases, antidepressants, for at least 8 weeks before entering the study. The class of drug patients at the time of the assessment was determined. The severity of depressive and manic residual symptoms, if present, was evaluated by a certified psychiatrist through the Hamilton Depression Rating Scale (HAM-D) [40] and the Young Mania Rating Scale (YMRS) [41]. All patients were required to be euthymic for at least 4 weeks prior to the neuropsychiatric and neuropsychological evaluation, showing HAM-D ≤ 7 and YMRS ≤ 12 total scores.

### Clinical Assessment and Neuropsychological Evaluation Paradigm

The following socio-demographic and clinical data of the sample were collected: age, gender, age of onset, years of education, medical and psychiatric comorbidity, family history for psychiatric disorders, duration of untreated illness (DUI), current treatment, lifetime number of manic/hypomanic/mixed and depressive episodes.

The assessed cognitive functions were: visual and selective attention, executive functioning, mental flexibility, verbal fluency and working memory. In particular, all patients and controls underwent a cognitive examination held by a professional neuropsychologist (M.C.) involving eight specific tasks: Stroop test -interference and errors- [42, 43], Tower of London -score- [44, 45], Span Attention test (Backward and Forward), Raven Matrices (score/time), Trail Making Task (partial score A, B and B-A) [46], Verbal Fluency Test (Category and Letter), Wisconsin Card Test (total score/error/perseverance/global) [47, 48], N-Back task (0-Back, 2-Back, 3-Back and Reaction Time) [49].

### Statistical Analysis

Descriptive analyses on socio-demographic and clinical data were performed using SPSS software, version 21.0. A one-way ANOVA was performed, comparing main and partial scores of each test in bipolar patients vs HCs. A further analysis was performed splitting patient sample into BD I and BD II groups (BD I vs BD II vs HCs). A *post hoc* test with Bonferroni correction of the level of significance was performed, taking into account that multiple comparisons have been performed with the same data, in order to assess possible differences among diagnostic subgroups.

## RESULTS

### Socio-Demographic and Clinical Findings

Main socio-demographic and clinical data of the study sample are summarised in (Table **1**).

The sample consisted of 15 patients with BD I (53% males; mean age 34.3 ± 10.3 SD years), 15 patients with BD II (54% males; mean age 37.3 ± 8 SD years) and 32 HCs (55.5% males; mean age 29.4 ± 10.6 SD years).

Bipolar patients and HCs were homogeneous in terms of gender ratio, age and years of education.

Approximately half of the patient sample had a positive family history for psychiatric disorders, that was higher in the BD II subgroup (66% vs 46% of BD I). BD II patients had twice the rate of psychiatric comorbidity compared to BD I subjects (53% vs 26%), medical comorbidity being only slightly higher in the same group (40% vs 33%). While the age of onset was comparable between BD I and II patients (23.6 vs 24.2 years), the duration of untreated illness (DUI) was significantly lower in the BD I group (13.7 months vs 40.6 months; F=8.503, p<.001). Depressive episodes were more common in BD II patients (7 vs 4), though not to a statistically significant level, while there were no differences in terms of manic/hypomanic/mixed episodes between the two subgroups. Regardless of polarity, the mean duration of the last episode was not statistically different between the two subgroups. Finally, concerning the treatment, the 20% of the sample was treated with a monotherapy, regardless of diagnosis, polytherapy being definitively more common within the bipolar patients’ sample.

### Neuropsychological Findings

Results of the one-way ANOVA comparing bipolar patients and HCs in terms of score performances are summarized in (Table **2**).

With respect to the attentional task, the Span Attention Test showed that bipolar patients obtained a lower score both at the Forward (5.8 vs 6.5) and the Backward mode (4.9 *vs* 5.5), that turned out to be statistically significant for the Forward mode (p<.001), with a borderline statistical significance at the Backward mode (p=.06). Results of the Span Attention Test are represented in (Fig. **1a**).

The Verbal Fluency Test showed a significantly lower performance at the Category score in the BD group (44.6 *vs* 51.1, p<.001). The Letter score of BD patients was lower than HCs, showing a trend of statistical significance (p=.08). Fig. (**1b**) shows the results of the Verbal Fluency Test.

With regard to the working memory task, the N-Back total score and 2-Back score were found to be significantly lower in BD patients compared to HCs (p respectively <.001 and .05). The score at 3-Back performance was lower in bipolar patients with a borderline statistical significance (p=.06) compared to HCs. Results of the Working Memory task are represented in Fig. (**1c**).

Matrices total score and Trail Making Task (B-A test) showed a coherent trend, being lower in bipolar patients, though not to a statistically significant level (p=.08).

After dividing the bipolar sample into subgroups, BD I and II patients showed significantly lower scores at Span (Forward) Test (p<.001) compared to HCs. Considering *post hoc* Bonferroni analysis, BD-I patients had a significantly lower score at Verbal Fluency Test (Category) compared to BD II and HCs (p<.001); conversely, at the 2-Back Task, BD-II obtained lower scores compared to BD I and HCs (p<.001).

## DISCUSSION

To date and to authors’ knowledge, this is the first study exploring cognitive functioning in patients with BD vs HCs, with a specific comparison of BD I vs II patients. In addition, the sample of bipolar patients was selected focusing on subjects under the age of 50 (mean age of 36 years), euthymic for at least 8 weeks and during a stable pharmacological treatment. The decision to exclude senior patients was taken in order to minimize a possible bias occurring with the inclusion of patients potentially suffering from precocious and not yet diagnosed mild cognitive impairment, caused by other neurological and vascular conditions.

Data on the characterization of the sample mostly reflected literature findings, with the only exception of age of onset that was similar in the two groups, being usually lower in BD I individuals [50]. Otherwise, socio-demographic and clinical features and treatment related variables were substantially consistent with what commonly reported from studies in the field. The mean duration of untreated illness was not surprisingly lower in subjects showing more severe and disturbing symptoms, as usually happens in BD I subjects due to manic episodes [51].

Several studies reported the presence of cognitive deficits in euthymic bipolar patients [52]. More in detail, impairment in executive functions (in particular category fluency and mental manipulation), memory (in particular immediate and delayed verbal memory) and attention are the most consistently reported findings in the field [52]. Some authors confirmed the presence of persistent neurocognitive deficits not only in the euthymic phase of BD but also in bipolar patients with alcohol abuse, suggesting an adjunctive negative effect on memory and frontal executive functions in bipolar individuals with this specific pattern of comorbidity [53].

In this perspective, our study supports previous findings and the notion of residual cognitive symptoms in euthymic bipolar patients, showing a statistically significant impairment in the Forward Attention Task, in the Category Fluency and in the Working Memory (n-Back and 2-Back test), compared to HCs. Nonetheless, it has to be noticed that, in the present study, cognitive deficits were detected only in a part of the administered tests, with some statistical values of borderline significance, suggesting that other cognitive functions resulted preserved. Concerning the possible confounding effect represented by comorbid alcohol abuse, we could exclude any interference caused by concomitant abuse, as this was an exclusion criterion, but we did not assess lifetime comorbidity with alcohol abuse.

More in detail, attention represents the ability to focus on a specific task. When this function is impaired (as we found in bipolar patients who had impairment in Forward Attention Task) patient are easily distracted. Attention is a basic requirement for being able to perform other cognitive tests and, when this function is impaired, the results of other tests may be hardly interpreted. Verbal fluency requires both language and executive skills; common assessment techniques usually include asking patients to list as many things in a category (Category) or as many words beginning with a specific letter (Letter) as they can, in a 1 minute period. The consequence of an impairment at these tests might be represented by an altered structure of the language, which may be poor and, sometimes, difficult to understand. An impaired working memory, as underlined by the worse results at N-Back test, indicates bipolar patients less able to plan strategies and organize solutions to everyday life problems.

Taken as a whole, these findings might be useful to guide research on specific cognitive domains potentially impaired in BD, memory and attention, in particular, given that our results were obtained from a group of young adults. In addition, reported results may be particularly useful in order to assess the epoch of onset of cognitive symptoms in bipolar patients. In fact, it has been reported that cognitive dysfunction has a significant impact on clinical outcome [11, 12,]. Hence, early identification and treatment of these symptoms are important in order to improve patients’ overall prognosis and quality of life.

Cognitive alterations in some patients with BD are evident not only during the acute phase of the disease: some studies, in fact, found cognitive impairment even before the onset [54, 55]. Furthermore, other studies revealed the presence of specific cognitive deficits also in patient’s relatives [24, 25, 56, 57], stressing the heritability of BD. The present study, however, did not include a sample of relatives of bipolar patients, being this issue, for future investigation, of particular relevance.

Another aim of the present study was to identify possible differences between the two subgroups of bipolar patients (BD I and BD II), being the argument largely neglected by the available literature. In this regard, only minor statistically significant differences were found in some tasks of cognitive assessment when comparing bipolar subgroups and HCs. In particular, BD I patients were found to perform worse on cognitive domains related to Verbal Fluency (*i.e.*, Verbal Fluency test, Category), while BD II subjects showed a worse performance in relation to Working Memory (*i.e.*, 2-Back Task).

These findings may suggest that cognitive dysfunctions may be present at the same level in both subtypes of BD, supporting the notion that BD II subjects do not simply suffer from a milder form of BD, as recently pointed out in European and American studies [58, 59]. Nonetheless, the hypothesis that euthymic BD I and II subjects may additionally show specific differences in terms of cognitive impairment remains to be further tested within larger sample studies.

The following limitations should be kept into consideration in the interpretation of reported results. The main limitation of the study is represented by the limited sample, particularly in the comparison of the diagnostic subsamples. This aspect reflected the screening process that brought to select only 30 subjects from 65 initial patients. Some of the reported results showing a borderline significance, in fact, may have shown more robust differences with larger samples. For future research, extending the assessment with follow-up observation of cognitive evaluations of patients, especially in the euthymic phases, would be of great clinical interest, in order to assess whether the duration of intercritical periods could influence the cognitive profile of bipolar patients. Literature, in fact, suggests the persistence of cognitive impairment in the euthymic phases of BD for some patients. However, it is not possible to exclude that a longer period of euthymia may lead to an improvement of the cognitive profile, with specific investigation in the field highly necessary.

Another bias may be represented by the lack of IQ assessment in patients and HCs, with the number of years of education used as an indirect evidence of the homogeneous intellective level of the sample. Furthermore, a potential influence of concomitant pharmacological treatment over cognitive functioning between bipolar patients and HCs cannot be ruled out. In fact, all patients were taking psychotropic medications, which could affect cognitive performance in the tests. Furthermore, with regard to the pharmacological treatment, we only recorded the class of psychotropic compounds, without collecting nor analyzing the potential effect of any specific molecule, which might, in turn, interfere with patient’s cognitive functioning. Finally, the sample was selected among patients attending a tertiary Clinic, specialised in the diagnosis and treatment of BD in Italy, and such sample may not be necessarily representative of the entire population of bipolar patients worldwide.

### CONCLUSION

In conclusion, the present study pointed out the presence of specific cognitive deficits in adult euthymic patients with BD compared with HCs, with marginal differences between bipolar I and II subjects. Further studies with larger samples are needed to confirm present results and explore the epoch of onset, the associated clinical features and the outcome influence of reported cognitive deficits in bipolar patients.

## Figures and Tables

**Fig. (1a):**
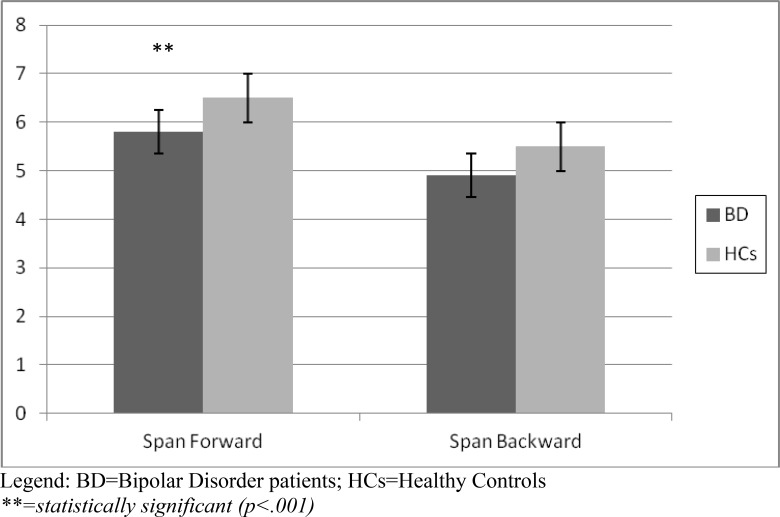
Span Attention Test (Forward and Backward) in BD patients *vs* HCs.

**Fig. (1b):**
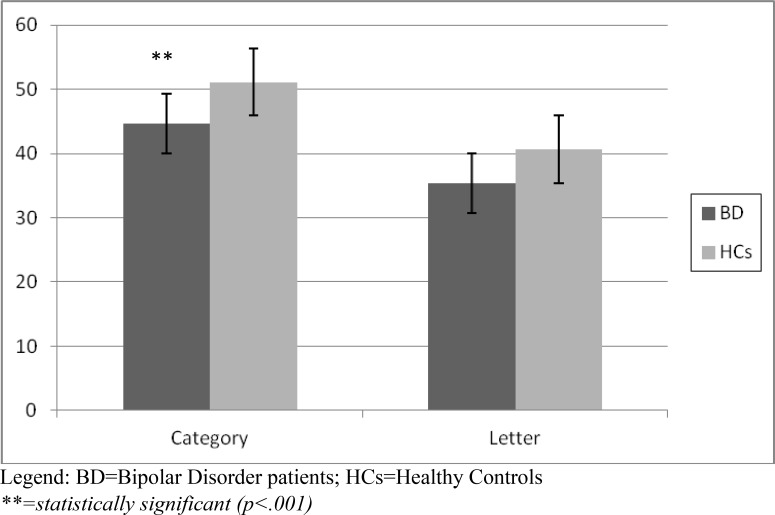
Verbal Fluency Task performance; Category and Letter task in BD patients *vs* HCs.

**Fig. (1c):**
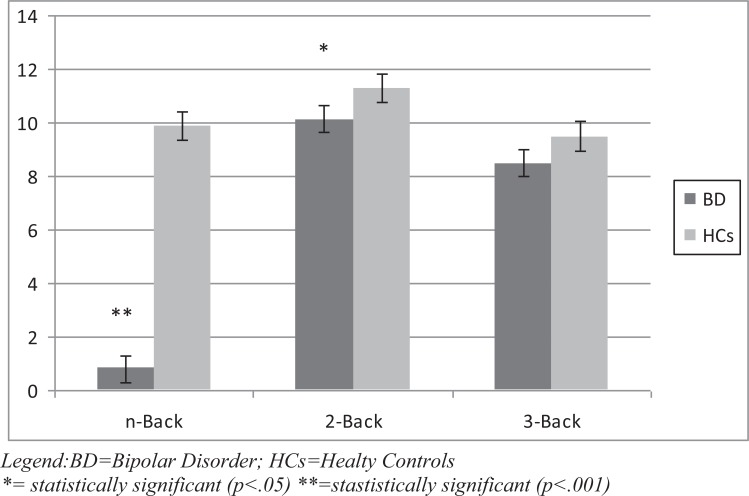
Working Memory: n-Back, 2-Back and 3-Back performance in BD patients *vs* HCs.

**Table 1:** Main socio-demographic and clinical characteristics of the total sample and diagnostic subgroups.

	BD (n=30)	BD I (n=15)	BD II (n=15)	HCs (n=32)	P value
Gender (F:M)	11:19	6:9	5:1	14:18	0.1
Age (m + SD)	36.1 (+10.1)	34.3(+10.3)	38.0(+9.6)	31.2(+11.8)	0.4
Education in years (m + SD)	14.3 (+1.6)	14.5(+1.9)	14.0(+1.6)	14.9(+2.1)	0.3
Age of onset (m + SD)	23.9(+6.8)	23.6(+5.8)	24.2(+7.9)	-	0.8
Psychiatric comorbidity (%)	40.0	26.6	53.3	-	0.1
Medical comorbidity (%)	36.6	33.3	40.0	-	0.3
Positive family history for psychiatric disorders (%)	56.6	46.6	66.0	-	0.4
DUI (months) (m + SD)	26.2(+18.5)	13.7(+19.2)**	40.6(+50.7)	-	0.0004
Number of depressive episodes (m)	6.3	4.0	7.0	-	0.2
Number of manic/mixed/hypomanic episodes (m)	6.6	6.3	7.0	-	0.1
Duration of last episode (days)	32.7	38.6	26.2	-	0.2
Monotherapy (%) ADs MSs	20.0 0 100.0	20.0 0 100.0	20.0 0 100.0	-	0.3

Values for categorical and continuous variables are expressed as n (%) and mean ± SD, respectively.

Standard deviations for continuous variables are shown in brackets.

Statistics: **F=8.503; p<.001

Legend: BD=Bipolar Disorder¸ BD I = Bipolar Disorder I patients, BD II= Bipolar Disorder II patients, HCs=Healthy Controls, DUI=Duration of Untreated Illness; ADs=antidepressants; MSs=mood stabilizers (lithium, anticonvulsants and atypical antipsychotics)

**Table 2:** Results of the neuropsychological battery administered to patients with BD *vs* HCs.

TEST	BD (n=30)	HCs (n=32)	P value
Span Forward (m+SD)**	5.8(+0.96)	6.5(+0.84)	0.0002
Span Backward (m+SD)§	4.9(+1.1)	5.5(+1.2)	0.06
Verbal Fluency Test (categories) (m+SD)**	44.6(+9.3)	51.1(+9.6)	0.0009
Verbal Fluency Test (letter) (m+SD) §§	35.3(+13)	40.6(+10.4)	0.08
Trial Making Test (B-A) (m+SD) §§	86.6(+32.2)	75.3(+15.8)	0.08
Matrices Score (m+SD) §§	45.89(+4.9)	47.7(+2.8)	0.08
n-Back Total (m+SD)**	0.81(+0.12)	9.89(+0.08)	0.0004
2-Back Test (m+SD)*	10.13(+1.8)	11.28(+1.6)	0.01
3-Back Test (m+SD)§	8.5(+2.4)	9.5(+1.5)	0.06

Values for categorical and continuous variables are expressed as n (%) and mean ± SD, respectively.

Standard deviations for continuous variables are shown in brackets.

Statistics: One-way ANOVA *p<.05; **p<.001; §p= 0.06 (borderline level of statistical significance); §§p= 0.08 (trend level of statistical significance)

Legend: BD=Bipolar Disorder patients; HCs=Healthy Controls
